# Metacarpal shaft fixation: a biomechanical comparison of dorsal plating, lag screws, and headless compression screws

**DOI:** 10.1186/s12891-021-04200-0

**Published:** 2021-04-07

**Authors:** Felix G. E. Dyrna, Daniel M. Avery, Ryu Yoshida, David Lam, Simon Oeckenpöhler, Mark P. Cote, Elifho Obopilwe, Craig M. Rodner, Augustus D. Mazzocca

**Affiliations:** 1 Department of Trauma, Hand and Reconstructive Surgery, University Hospital Münster, Albert-Schweitzer-Campus 1, Building W1, 48149 Münster, Germany; 2 OrthoSports Associates Upper Extremity Surgeon, Birmingham, AL USA; 3 Department of Orthopaedic Surgery, University of Connecticut, Farmington, CT USA

**Keywords:** Metacarpal fracture, Headless compression screw, Intramedullary screw fixation, Biomechanics

## Abstract

**Background:**

Metacarpal shaft fractures are common and can be treated nonoperatively. Shortening, angulation, and rotational deformity are indications for surgical treatment. Various forms of treatment with advantages and disadvantages have been documented. The purpose of the study was to determine the stability of fracture fixation with intramedullary headless compression screws in two types of metacarpal shaft fractures and compare them to other common forms of rigid fixation: dorsal plating and lag screw fixation. It was hypothesized that headless compression screws would demonstrate a biomechanical stronger construct.

**Methods:**

Five matched paired hands (age 60.9 ± 4.6 years), utilizing non-thumb metacarpals, were used for comparative fixation in two fracture types created by an osteotomy. In transverse diaphyseal fractures, fixation by headless compression screws (*n* = 7) and plating (*n* = 8) were compared. In long oblique diaphyseal fractures, headless compression screws (*n* = 8) were compared with plating (*n* = 8) and lag screws (*n* = 7). Testing was performed using an MTS frame producing an apex dorsal, three point bending force. Peak load to failure and stiffness were calculated from the load-displacement curve generated.

**Results:**

For transverse fractures, headless compression screws had a significantly higher stiffness and peak load to failure, means 249.4 N/mm and 584.8 N, than plates, means 129.02 N/mm and 303.9 N (both *p* < 0.001). For long oblique fractures, stiffness and peak load to failure for headless compression screws were means 209 N/mm and 758.4 N, for plates 258.7 N/mm and 518.5 N, and for lag screws 172.18 N/mm and 234.11 N. There was significance in peak load to failure for headless compression screws vs plates (*p* = 0.023), headless compression screws vs lag screws (*p* < 0.001), and plates vs lag screws (*p* = 0.009). There was no significant difference in stiffness between groups.

**Conclusion:**

Intramedullary fixation of diaphyseal metacarpal fractures with a headless compression screw provides excellent biomechanical stability. Coupled with lower risks for adverse effects, headless compression screws may be a preferable option for those requiring rapid return to sport or work.

**Level of evidence:**

Basic Science Study, Biomechanics.

## Background

Metacarpal fractures account for almost 18% of all fractures distal to the elbow, with non-thumb fractures accounting for 88% of this group [1]. Metacarpal shaft fractures can be treated nonoperatively, but shortening, angulation, and rotational deformity can diminish clinical function [2]. As such, surgical stabilization is commonly employed to restore anatomic alignment.

Current methods for metacarpal shaft fixation include Kirschner wires (k-wires), plating, and screws, depending on fracture orientation and physicians preference. Plating represents a strong construct for a wide variety of fracture types with the drawbacks of more extensive soft tissue dissection, higher infection risk, risk of extensor adhesions, or hardware irritation [3, 4]. Screws represent another rigid form of fixation and are an option with long oblique or spiral fractures. Although less rigid, percutaneous K-wires are appealing as they decrease soft tissue dissection. A disadvantage of k-wires is that they protrude through the skin risking infection and making early range of motion difficult [5].

With the growing active population, alternate forms of fixation to decrease tissue dissection, lower infection risk, lower risk of extensor tendon adhesion, lower hardware irritation, and allow early active motion and rapid return to function deserve consideration. Headless compression screws in hand fractures have emerged in a few clinical studies showing good results for function and healing with the advantages listed above [6–8].

The purpose of the study was to determine the stability of fracture fixation with intramedullary headless compression screws in two types of metacarpal shaft fractures and compare them to other common forms of rigid fixation: dorsal plating and lag screw fixation. It was hypothesized that headless compression screws would demonstrate higher load to failure than plates in transverse fractures and higher load to failure than plates and lag screws in long oblique fractures.

## Methods

### Specimen Preparation & Fixation

Five matched paired hands, mean age 60.9 ± 4.6 years, were obtained and underwent dual energy x-ray absorptiometry (DEXA) (Lunar DPI XQ dexascan Madison, Wis). A 1cm^2^ region within the metacarpal neck was measured. Metacarpals were dissected free, excluding the thumb metacarpals, yielding 40 metacarpals. Two metacarpals were disqualified from use due to previous deformity. Diaphyseal fractures were created with an oscillating saw, one millimeter thick. In fifteen, the osteotomies were transverse located 25 mm from the distal articular surface, and in twenty-three were long oblique with the osteotomy beginning dorsally 20 mm from the distal articular surface and exiting 35 mm palmarly. In the first group of matched paired hands of transverse fractures, metacarpals were fixated with dorsal non locking plating (*n* = 8) versus metacarpals with headless compression screws (*n* = 7). The second group of match paired hands with long oblique fractures fixated with dorsal non locking plating (*n* = 8) versus headless compressions screws (*n* = 8) versus two lag screws (*n* = 7).

**Fig. 1:**
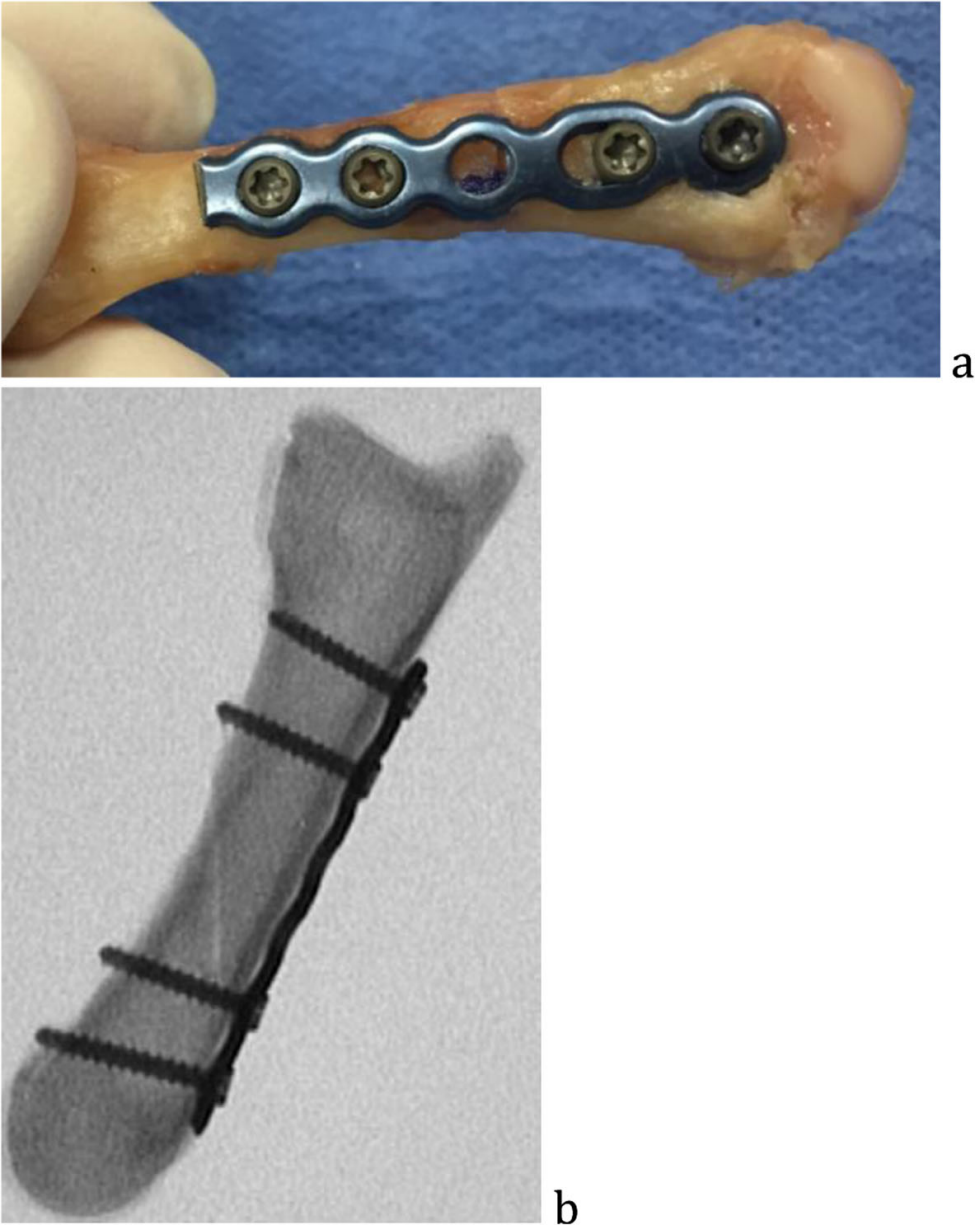
Plate and screw construct, gross specimen (**a**) in transverse osteotomy and lateral fluoroscopic image in oblique osteotomy (**b**)

In the dorsal plating groups, low profile 2.4 mm plates (Arthrex, Naples, Florida) were placed on the dorsal surface. The transverse fractures utilized a six-hole plate, and for the long oblique fractures seven-hole plates. In each, two 2.4 mm non-locking screws were inserted bicortically on each side (Fig. 1). There has not been sufficient evidence for the need of three screws on each fracture side [9].

**Fig. 2:**
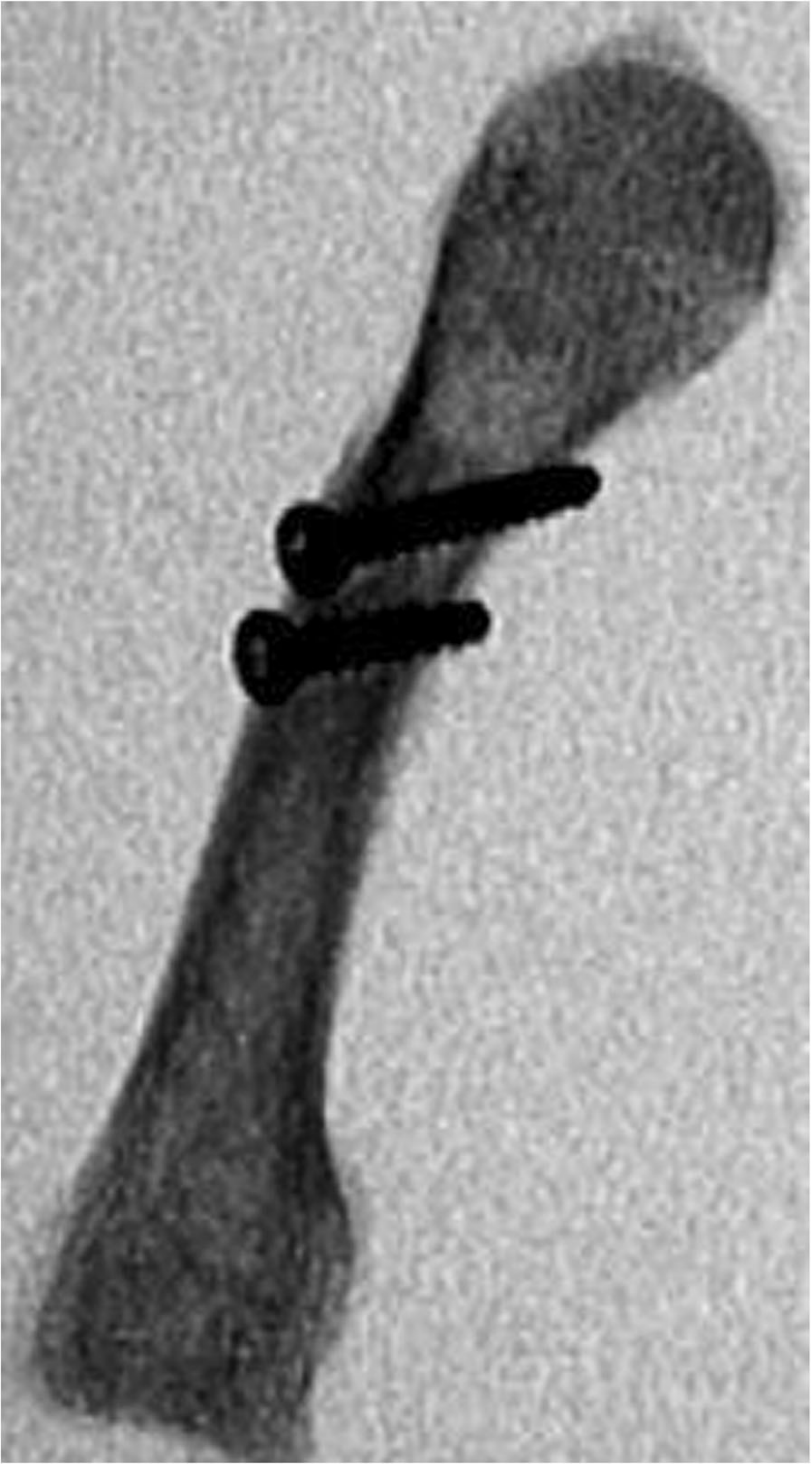
Lateral fluoroscopic image of lag screw construct in oblique osteotomy

In the lag screw fixation, two 2.4 mm non-locking cortical screws (Arthrex, Naples, Florida) were inserted perpendicular to the fracture line. Each near cortex was drilled with a 2.4 mm drill bit, and the far cortex with a 1.7 mm drill bit, according to standard lag screw technique (Fig. 2).

**Fig. 3:**
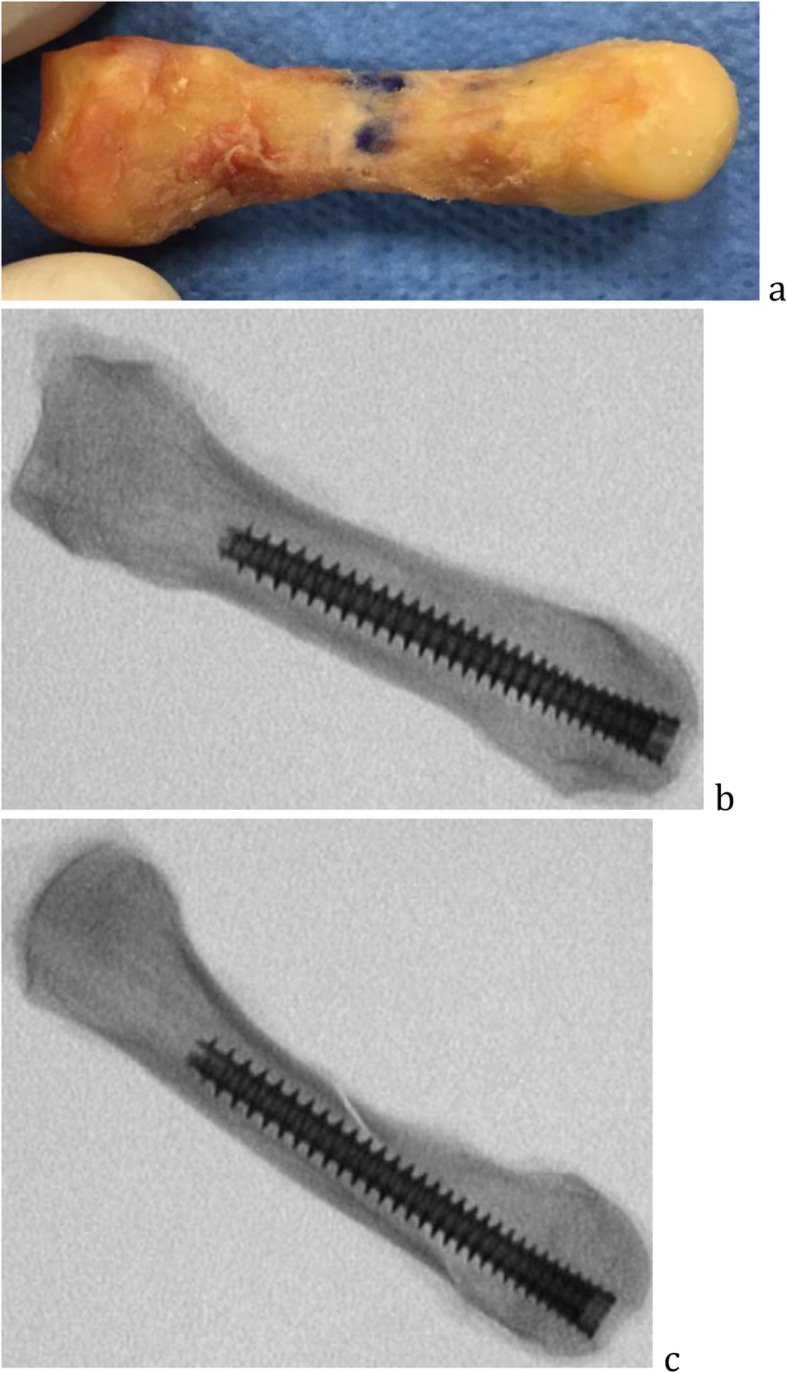
Headless compression screw fixation, gross specimen (**a**), anteroposterior in transverse osteotomy (**b**), and lateral in oblique osteotomy fluoroscopic imaging (**c**)

In the headless compression screw group, a 1.1 mm guidewire was inserted into dorsal third articular surface of the metacarpal head and subsequently into a central position of the metacarpal shaft on anteroposterior and lateral fluoroscopic imaging. The distal metacarpal articular surface was then opened with a 2.4 mm cannulated drill bit if using a 3.5 mm compression screw, and a 3.2 mm cannulated drill bit if using a 4.0 mm cannulated screw. The cannulated headless compression screw (Arthrex, Naples, Florida) was inserted over the guidewire to a depth of approximately 2 mm below the closest (dorsal) articular surface. Small and ring metacarpals were fixated with the 3.5 mm compression screw and long and index with 4.0 mm compression screws based on the diameter of the medullary canal (Fig. 3).

### Testing protocol

**Fig. 4:**
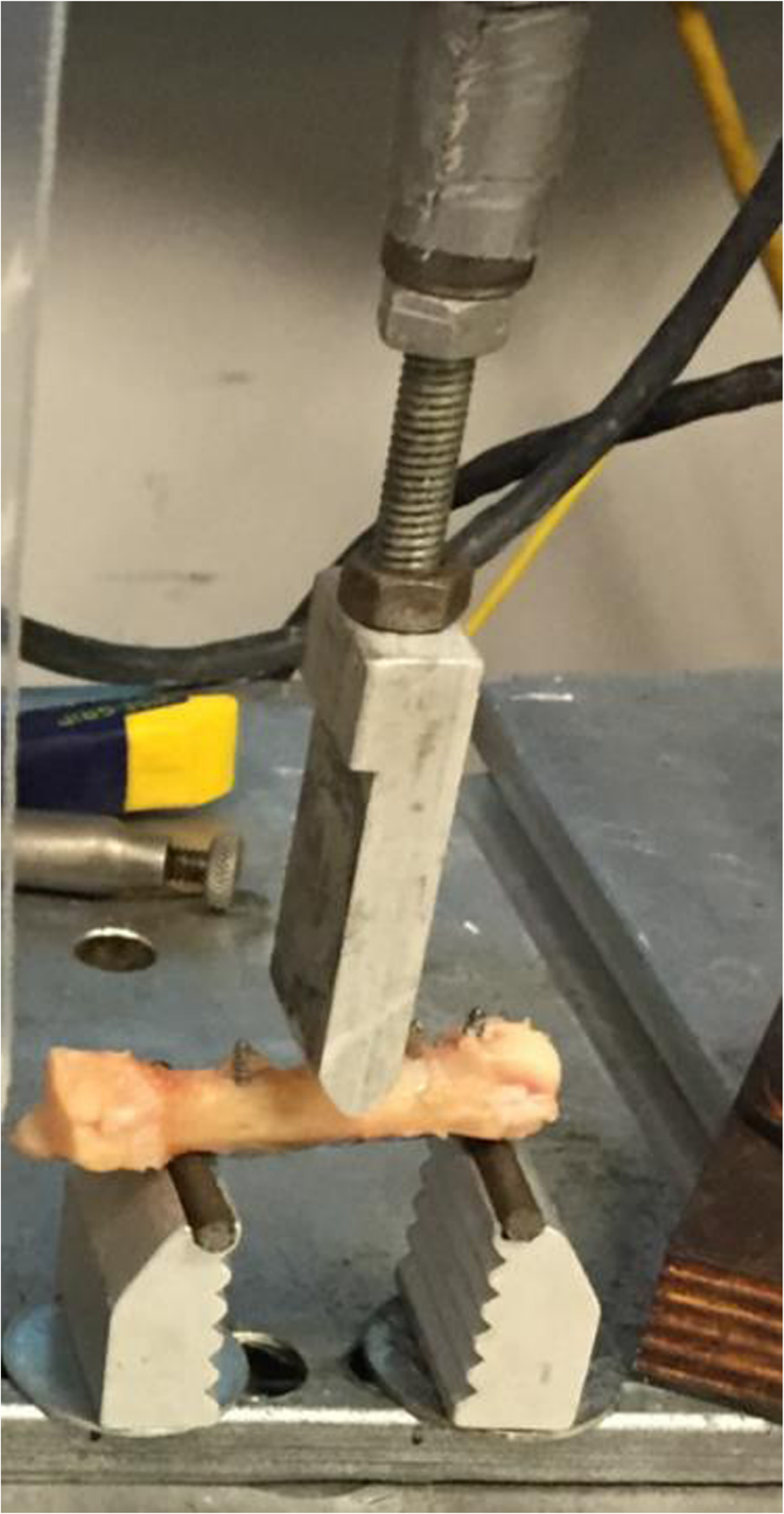
Testing setup with loading pin centered over osteotomy producing an apex dorsal force on the MTS servohydraulic frame

Each metacarpal was subjected to a three point bending, apex dorsal force, on a Materials Testing System (MTS) servohydraulic test frame (MTS Systems Corp, Eden Prairie, MN), similar to previous studies [5, 10, 11]. Each sample was placed with the dorsal surface downward toward the base plate and the loading pin centered over the fracture site (Fig. 4). The loading pin displaced toward the MTS actuator at a rate of 100 mm/min until failure. Sampling of the load displacement was recorded at a rate of 100 Hz. Failure was defined by sudden change in the load-displacement curve.

### Data analysis

Load displacement curves were generated for each specimen. The curve was utilized to calculate the peak load to failure (N) and stiffness (N/mm). The peak load to failure was the maximum force at which an acute change in the load-displacement curve was observed. Stiffness of the construct corresponded to the slope of the load-displacement curve before the peak load was reached.

### Statistics

Given the variation of testing specimens in previous metacarpal biomechanical studies [1, 5, 10, 12], an a priori power analysis was difficult. We assumed the smallest difference would be between the headless compression screws and plate fixation groups. A difference of 75 N between these groups was selected as the threshold for a relevant difference in load to failure. A standard deviation of ±50 N was assumed across groups. Assuming these parameters, a sample size of 7 per group would provide 80% power to detect a 75 N difference in load to failure at an alpha level of 0.05.

Descriptive statistics to categorize the groups were calculated using mean and standard deviation. Differences between the groups in load to failure and slope were tested with one way ANOVA with a post hoc Bonferroni correction where appropriate for longitudinal fractures and an independent *t* test for transverse fractures. A *p* value less than 0.05 was considered statistically significant.

## Results

### Transverse shaft fracture

**Fig. 5:**
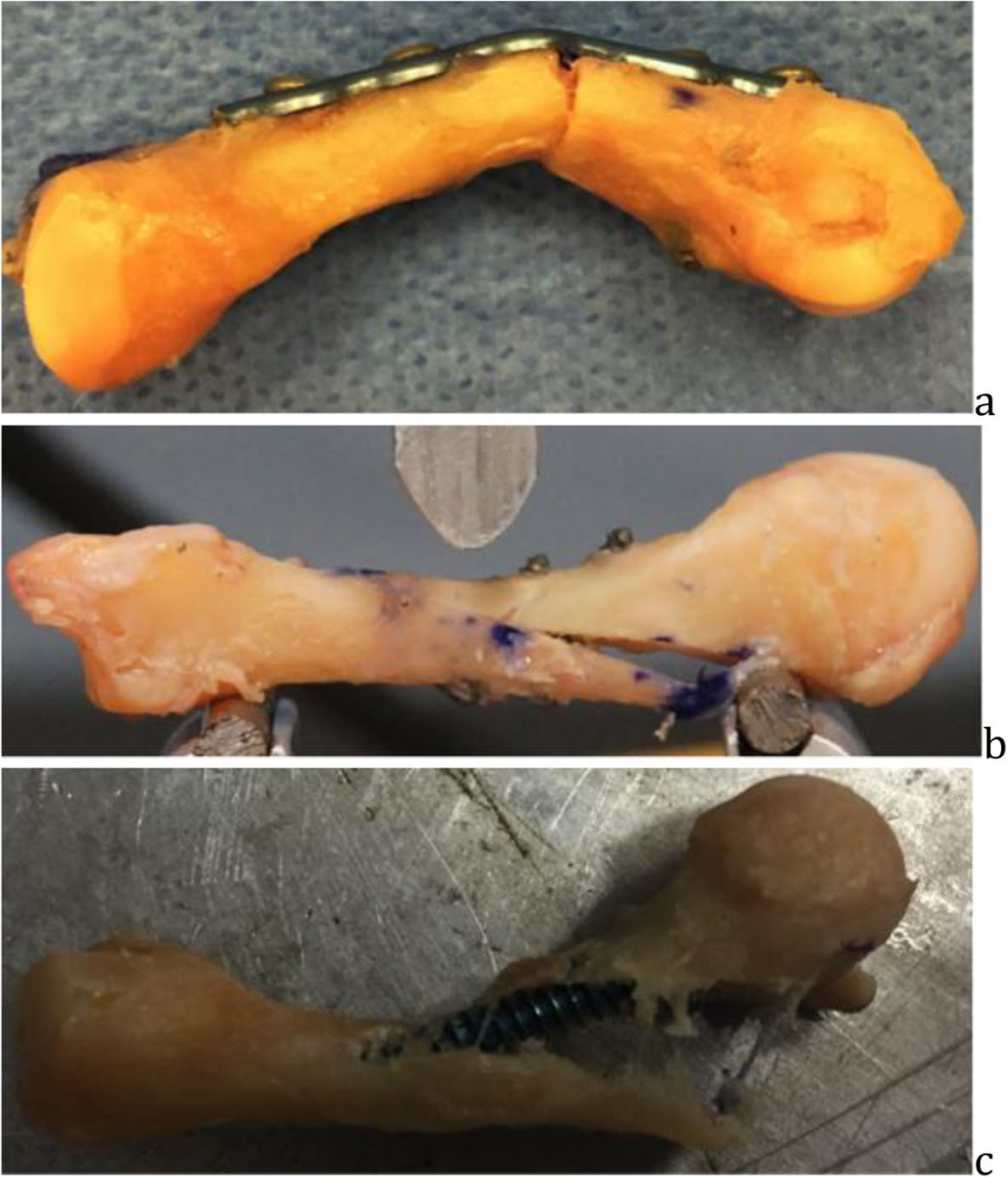
Failure patterns, **a** plate bending in a transverse osteotomy, **b** lag screw disengagement in oblique osteotomy, and **c** headless compression screw loosening in metacarpal head and displacement at osteotomy site of oblique pattern

For plate and screw constructs, the mean peak load to failure was 303.9 ± 94.47 N and mean stiffness was 129.02 ± 66.18 N/mm. All specimens failed by plate bending (Fig. 5a).

Headless compression screws fixation showed a mean peak load to failure of 829.8 ± 116.4 N and mean stiffness was 316.9 ± 101.6 N/mm. Four specimens failed by the screw bending and three specimens failed by loosening in the metacarpal head region with resultant displacement at the fracture site.

A significant difference was observed in both peak load to failure (*p* < 0.001) and stiffness (*p* < 0.001) in favor of the headless compression screw group. There was no significant difference in bone mineral density between headless compression screw and plate specimens (*p* = 0.626).

### Oblique shaft fractures

In plate and screw constructs, the mean peak load to failure was 518.5 ± 212.45 N and mean stiffness was 258.7 ± 99.68 N/mm. All specimens failed by plate bending.

In two lag screw constructs, the mean peak load to failure was 234.11 ± 178.06 N and mean stiffness was 172.18 ± 122.28 N/mm. All specimens failed by screw heads pulling through the near (dorsal) cortex (Fig. 5b).

For headless compression screw constructs, the mean peak load to failure was 758.4 ± 54.9 N and mean stiffness was 275.1 ± 62.6 N/mm. All specimens failed by loosening of the implant in the metacarpal head region and displacement at the fracture site (Fig. 5c).

Comparison of the groups showed a significant difference in peak load to failure of headless compression screws versus plate and screws (*p* = 0.023), headless compression screw versus lag screws (*p* < 0.001), and plate and screws versus lag screws (*p* = 0.009). A significant difference in stiffness was not observed between groups (*p* = 0.12). There were no significant differences in bone mineral density between specimens of headless compression screw and plate (*p* = 0.42), plate and lag screws (*p* = 0.46), or headless compression screw and lag screws (*p* = 0.94).

## Discussion

In the current study, biomechanical stability of various fixation methods for diaphyseal metacarpal fractures were compared. In transverse fractures, intramedullary headless compression screw was compared to dorsal plating. The former was found to be superior in both stiffness and peak load to failure. In the oblique fractures, intramedullary headless compression screw, dorsal plating, and two lag screws were compared. While the intramedullary compression screw had significantly higher peak to load failure compared to the other two methods, there was no significant difference in stiffness.

Cannulated headless compression screws have been widely used in hand surgery since Herbert and Fisher [13] introduced its utility in scaphoid fractures. However, its use as an intramedullary device for metacarpal fractures is relatively new [6, 14, 15].

The intramedullary compression screw has several advantages over conventional fixation methods. It avoids the risk of pin tract infection or need for removal associated with percutaneous K-wire fixation. Unlike dorsal plating which requires a more extensive dissection, the intramedullary screw can be inserted through a small dorsal incision. Furthermore, with the compression screw, the entire hardware is buried inside the metacarpal and thus unlikely to cause irritation of surrounding soft tissue or tendon ruptures. Finally, the compression screw allows for early active motion exercises to minimize stiffness when compared to K-wires [7, 8, 16]. These advantages make intramedullary compression screw an attractive option for fixation of metacarpal fractures. Some downsides of intramedullary screw fixation have to be mentioned on the other hand as extensor tendon disruption and difficult explantation in case of infection or refracture is possible [10, 17].

The results from the current study support that compression screw fixation provides excellent biomechanical stability that is comparable to dorsal plating or lag screws. Analogous to plate fixation, it may provide an option for rapid return to sport [18] with a lower risk of adverse effects. All construct exceeded by far the invivo forces generated by the flexor tendons of 30 N and therefore can be considered possible options [19] from a biomechanically point of view.

In comparison to existing biomechanical data by Oh et al. we could demonstrate higher peak loads for all tested devices [20]. This may be due to the effect of a more stable plate and larger diameter intramedullary compression screws with a different design. Especially the size of the intramedullary compression screw has to fit perfectly in order to generate optimize fixation strength. Oftentimes the available screw size may not be large enough to archive endosteal purchase [21].

Reported clinical results for compression screw fixation have been good. Del Pinal et al. [14] reported a series of 48 metacarpal fractures, all of which healed with total active motion (sum of distal phalangeal joint, proximal phalangeal joint, and metacarpophalangeal joint motion) averaging 249 degrees. Ruchelsman et al. [6] first reports a series of 20 metacarpal fractures in which all fractures healed with full flexion and grip strength measuring 105% (range 58 to 230%) compared to the contralateral side. In their second series of now 91 patients they could continue to show favorable results without the disadvantages of open reduction and fixation [7].

There are several limitations of the study. Failure of the constructs was tested only in one dimension and without cyclic loads demonstrating a possible loosening during the early postoperative phase. We chose to load the construct with an apex-dorsal force based on previous biomechanical studies [5, 10, 11]. Axial, rotational, or cyclical loading may reveal clinically important differences. Also, only 3-point bending instead of cantilever bending was used that may not reflect individual anatomy and physiology of the hand and metacarpal loading.

Particularly with plate fixation, other methods or construct configurations are possible and are not accounted for, such as locking plate constructs, which could demonstrate different results. Additionally, the current study was performed on cadavers and does not account for any differences in biological healing the different constructs induce. However, differences in bone mineral density between cadaver specimens was tested and found to be insignificant.

It should also be noted that the retrograde placement of intramedullary screw requires an arthrotomy of MCP joint as it is inserted through the articular surface of the metacarpal head which may put the joint at an increased risk for future arthritis [22]. Furthermore perfect anatomic reduction may be more difficult percutaneously than in open techniques, but clinical results remain good [6, 7, 14].

## Conclusion

Intramedullary fixation of diaphyseal metacarpal fractures with a headless compression screw provides excellent biomechanical stability. Coupled with lower risks for adverse effects, headless compression screws may be a preferable option for those requiring rapid return to sport or work.

## Data Availability

The datasets generated and analyzed during the current study are not publicly available, but are available as de-identified data sheet from the corresponding author on reasonable request.

## Abbreviations

ANOVA: Analysis of variance
MCP: Metacarpophalangeal

## References

[1] Curtis BD, Fajolu O, Ruff ME, Litsky AS (2015). Fixation of Metacarpal Shaft Fractures: Biomechanical Comparison of Intramedullary Nail Crossed K-Wires and Plate-Screw Constructs. Orthop Surg.

[2] McCarthy C, Samora JB, Awan H (2014). Metacarpal shaft fractures: a review. OA Orthopaedics.

[3] Weinstein LP, Hanel DP (2002). Metacarpal fractures. J Am Soc Surg Hand.

[4] Fusetti C, Meyer H, Borisch N, Stern R, Santa DD, Papaloïzos M (2002). Complications of plate fixation in metacarpal fractures. J Trauma.

[5] Hiatt SV, Begonia MT, Thiagarajan G, Hutchison RL (2015). Biomechanical comparison of 2 methods of intramedullary K-wire fixation of transverse metacarpal shaft fractures. J Hand Surg Am.

[6] Ruchelsman DE, Puri S, Feinberg-Zadek N, Leibman MI, Belsky MR (2014). Clinical outcomes of limited-open retrograde intramedullary headless screw fixation of metacarpal fractures. J Hand Surg Am..

[7] Eisenberg G, Clain JB, Feinberg-Zadek N, Leibman M, Belsky M, Ruchelsman DE (2019). Clinical Outcomes of Limited Open Intramedullary Headless Screw Fixation of Metacarpal Fractures in 91 Consecutive Patients. Hand (New York, N,Y).

[8] Beck CM, Horesh E, Taub PJ (2019). Intramedullary screw fixation of metacarpal fractures results in excellent functional outcomes. Plast Reconstr Surg.

[9] Canton SP, Dadi S, Anthony A, Black RT, Clancy M, Fowler JR. Comparison of Screw Quantity and Placement of Metacarpal Fracture Fixation: A Biomechanical Study. Hand (New York, N,Y). 2020:1558944720974116. [Epub ahead of print] 10.1177/1558944720974116 PMC9465802 33349049

[10] Watt AJ, Ching RP, Huang JI (2015). Biomechanical evaluation of metacarpal fracture fixation: application of a 90° internal fixation model. Hand (New York, N,Y).

[11] Avery DM, Klinge S, Dyrna F, Pauzenberger L, Lam D, Cote M (2017). Headless Compression Screw Versus Kirschner Wire Fixation for Metacarpal Neck Fractures: A Biomechanical Study. J Hand Surg Am.

[12] Barr C, Behn AW, Yao J (2013). Plating of metacarpal fractures with locked or nonlocked screws, a biomechanical study: how many cortices are really necessary?. Hand (New York, N,Y).

[13] Herbert TJ, Fisher WE (1984). Management of the fractured scaphoid using a new bone screw. J Bone Joint Surg Br.

[14] del Piñal F, Moraleda E, Rúas JS, de Piero GH, Cerezal L (2015). Minimally invasive fixation of fractures of the phalanges and metacarpals with intramedullary cannulated headless compression screws. J Hand Surg Am..

[15] Borbas P, Dreu M, Poggetti A, Calcagni M, Giesen T (2016). Treatment of proximal phalangeal fractures with an antegrade intramedullary screw: a cadaver study. J Hand Surg Eur Vol.

[16] Warrender WJ, Ruchelsman DE, Livesey MG, Mudgal CS, Rivlin M. Low Rate of Complications Following Intramedullary Headless Compression Screw Fixation of Metacarpal Fractures. Hand (New York, N,Y.). 2020;15:798–804. 10.1177/1558944719836214 PMC7850257 30894028

[17] Labèr R, Jann D, Behm P, Ferguson SJ, Frueh FS, Calcagni M (2020). Intramedullary screw fixation for metacarpal shaft fractures: a biomechanical human cadaver study. J Hand Surg Eur Vol.

[18] Etier BE, Scillia AJ, Tessier DD, Aune KT, Emblom BA, Dugas JR (2015). Return to play following metacarpal fractures in football players. Hand (New York, N,Y).

[19] Schuind F, Garcia-Elias M, Cooney WP, An KN (1992). Flexor tendon forces: in vivo measurements. J Hand Surg Am..

[20] Oh JR, Kim DS, Yeom JS, Kang SK, Kim YT (2019). A comparative study of tensile strength of three operative fixation techniques for metacarpal shaft fractures in adults: a cadaver study. Clin Orthop Surg.

[21] Dunleavy ML, Candela X, Darowish M. Morphological Analysis of Metacarpal Shafts With Respect to Retrograde Intramedullary Headless Screw Fixation. Hand (New York, N,Y). 2020:1558944720937362. [Epub ahead of print] 10.1177/1558944720937362 PMC9274869 32666845

[22] ten Berg PWL, Mudgal CS, Leibman MI, Belsky MR, Ruchelsman DE (2013). Quantitative 3-dimensional CT analyses of intramedullary headless screw fixation for metacarpal neck fractures. J Hand Surg Am.

